# Repeat two-stage exchange arthroplasty for recurrent periprosthetic hip or knee infection: what are the chances for success?

**DOI:** 10.1007/s00402-021-04330-z

**Published:** 2022-01-04

**Authors:** A. C. Steinicke, J. Schwarze, G. Gosheger, B. Moellenbeck, T. Ackmann, C. Theil

**Affiliations:** Department of Orthopedics and Tumor Orthopedics, Muenster University Hospital, Muenster, Albert-Schweitzer-Campus 1, 48149 Muenster, Germany

**Keywords:** Periprosthetic joint infection, Reinfection, Revision arthroplasty, THA, TKA, Two-stage exchange arthroplasty

## Abstract

**Introduction:**

Two-stage revision is a frequently chosen approach to treat chronic periprosthetic joint infection (PJI). However, management of recurrent infection after a two-stage exchange remains debated and the outcome of a repeat two-stage procedure is unclear. This study investigates the success rates of repeat two-stage exchange arthroplasty and analyzes possible risk factors for failure.

**Materials and methods:**

We retrospectively identified 55 patients (23 hips, 32 knees) who were treated with repeat resection arthroplasty and planned delayed reimplantation for recurrent periprosthetic joint infection between 2010 and 2019 after a prior two-stage revision at the same institution. The minimum follow-up was 12 months with a median follow-up time of 34 months (IQR 22–51). The infection-free survival, associated revision surgeries, and potential risk factors for further revision were analyzed using Kaplan–Meier survival curves and comparative non-parametric testing.

**Results:**

78% (43/55) underwent reimplantation after a repeat implant removal. Of those who completed the second-stage surgery, 37% (16/43) underwent additional revision for infection and 14% (6/55) underwent amputation. The reinfection-free implant survivorship amounted to 77% (95% CI 64–89%) after 1 year and 38% (95% CI 18–57%) after 5 years. Patients with a higher comorbidity score were less likely to undergo second-stage reimplantation (median 5 vs. 3, *p* = 0.034). Furthermore, obese patients (*p* = 0.026, Fisher’s exact test) and diabetics (*p* < 0.001, log-rank test) had a higher risk for further infection. Most commonly cultures yielded polymicrobial growth at the repeat two-stage exchange (27%, 15/55) and at re-reinfection (32%, 9/28). Pathogen persistence was observed in 21% (6/28) of re-reinfected patients.

**Conclusion:**

The success rates after repeat two-stage exchange arthroplasty are low. Patients must be counseled accordingly and different modes of treatment should be considered.

## Introduction

Periprosthetic joint infection (PJI) is a severe complication of total joint arthroplasty and occurs in around 1–2% of primary knee or hip arthroplasties [1]. As the demand for total joint arthroplasty is on the rise due to an aging population, the revision burden due to PJI is expected to increase as well [1–3].

Two-stage exchange usually using an antibiotic-loaded spacer is considered the gold standard in treating chronic PJI [1]. A two-stage approach with removal of the implant and all foreign material, debridement, and irrigation during first-stage surgery allows for a thorough debridement of all infected tissue and can be considered appropriate for all chronic infection regardless of culture results prior to surgery, soft tissue conditions, and timing of the infection. However, despite these general advantages, two-stage revision surgery is associated with a great deal of morbidity due to the two surgeries required and the period in between stages and the rate of reinfection can be as high as 30% in some cases [4–10]. Recurrence of infection can be associated with further morbidity as well as a high mortality, particularly if further surgeries are needed [11–13]. One option in these cases is to perform a repeat two-stage procedure, i.e., another sequence of explantation, spacer insertion, and delayed reimplantation of the infected prosthesis [11–13]. Nonetheless, it is debated whether a repeat two-stage exchange is adequate, as poor infection-free survival has been reported with failure rates ranging from 22 to 49% [11–15]. When taken into consideration that many patients do not undergo second-stage reimplantation, failure rates might be even higher [16].

However, despite the high expected rate of reinfection, there is a scarcity of studies on the outcome of a repeat two-stage exchange and potential risk factors for failure remain unknown.

This study investigates the success rates of repeat two-stage procedures for hip and knee PJI at a single institution, analyzes microbiological findings, and describes possible risk factors for failure.

## Material and methods

The approval of the local ethics committee (2019-042-F-s Ethikkommission der Aerztekammer Westfalen-Lippe und der Westfaelischen-Wilhelms Universitaet Muenster) was obtained before initiation of this retrospective cohort study. Patients were included if they met the following criteria: history of a completed two-stage exchange arthroplasty for chronic hip or knee PJI at our institution, diagnosis of further periprosthetic joint infection of the same joint analog to the criteria published by the Musculoskeletal Infection Society (MSIS) from 2011 [17], treatment with repeat resection arthroplasty and planned delayed reimplantation at our institution between 2010 and 2019, and a minimum follow-up period of 1 year. However, patients who did undergo revision surgery or died prior to that were included [18]. Patients with prior resection of a bone tumor and subsequent infection were excluded from this study. Using our prospectively maintained institutional joint registry, we identified 305 patients who had undergone two-stage exchange arthroplasty of a hip or knee prosthesis due to chronic PJI at our institution between 2010 and 2017. Of these, 55 were treated with repeat resection arthroplasty and planned delayed reimplantation between 2010 and 2019 due to reinfection. The median follow-up period was 34 months (interquartile range (IQR) 22–51).

Success of the repeated two-stage exchange arthroplasty was defined following the Delphi-based consensus definition that includes healed wounds, no further surgical procedure for infection, and no PJI-related mortality [19].

Data regarding the patients’ surgical history, clinical course, medication, and preexisting comorbidities were collected from electronic files. An age-adjusted Charlson Comorbidity Index (CCI) was calculated for each patient [20]. Microbiological findings are presented for the initial two-stage infection, second two-stage, and potential further revisions. Infections were classified as persistent rather than new infections, if at least one pathogen that was cultured at the explantation stage of the preceding two-stage exchange arthroplasty was cultured again at the subsequent two-stage exchange or any further revision for PJI failure [21].

**Fig. 1:**
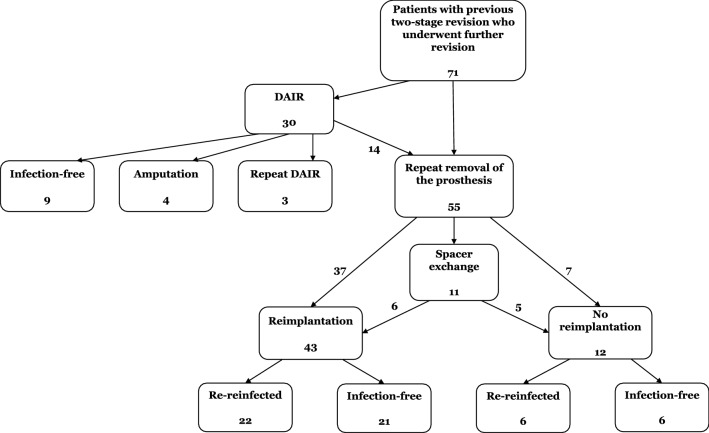
Flowchart of the treatment course and final outcome

Patient demographics of the study cohort at the time of the repeat two-stage exchange are showcased in Tables 1 and 2. The median age at the time of the repeat two-stage exchange was 73 years (IQR 64–78). An overview of the patients’ course and outcomes is provided in Fig. 1.

**Table 1:** Patient demographics for patients with or without further revision for reinfection after a repeat two-stage exchange

Variable	Entire study cohort	Reinfection	No reinfection	*p* (Fisher’s exact test)
Female	49% (27/55)	48% (13/27)	52% (14/27)	0.790
TKA	58% (32/55)	53% (17/32)	47% (15/32)	0.787
Diabetes mellitus	40% (22/55)	68% (15/22)	32% (7/22)	0.054
Obesity	64% (35/55)	63% (22/35)	37% (13/35)	**0.026**
Chronic kidney disease	27% (15/55)	53% (8/15)	47% (7/15)	1.000
Hypertension	85% (47/55)	51% (24/47)	49% (23/47)	1.000
Heart disease	51% (28/55)	57% (16/28)	43% (12/28)	0.423
Depression	25% (14/55)	50% (7/14)	50% (7/14)	1.000
COPD	18% (10/55)	50% (5/10)	50% (5/10)	1.000
Anticoagulation	29% (16/55)	38% (6/16)	63% (10/16)	0.245
Tobacco use	20% (11/55)	27% (3/11)	73% (8/11)	0.101
Additional DAIR before repeat two-stage revision	25% (14/55)	43% (6/14)	57% (8/14)	0.547

^*p*^ values for Fisher’s exact test

Significant differences are marked in bold

**Table 2:** Patient demographics for patients with or without further revision for reinfection after a repeat two-stage exchange

Variable	Entire study cohort	Patients reinfected	Patients not reinfected	*p* (Mann–Whitney *U* test)
Age at second PJI	73 (IQR 64–78)	72 (IQR 64–78)	73 (IQR 64–79)	0.655
Body mass index	31.4 (IQR 27.7–35.4)	32 (IQR 30–38)	29 (IQR 26–33)	**0.040**
Age-adjusted CCI	4 (IQR 3–5)	4 (IQR 3–5)	4 (IQR 3–5)	**0.314**
Months between first and second PJI	13 (IQR 4–32)	16 (IQR 5–35)	10 (IQR 2–26)	0.162
Number of previous surgeries before second PJI	5 (IQR 4–7)	5 (IQR 4–7)	5 (IQR 4–7)	0.939

^*p*^ values for Mann–Whitney *U* test

Significant differences are marked in bold

### Surgical approach

The first stage of a two-stage exchange arthroplasty included the thorough removal of all foreign material and bradytrophic tissue, irrigation, and if needed resection of osteomyelitic bone. Three to seven tissue samples were obtained and sent to our hospital’s institute for microbiology, where they were cultured for 7–14 days on Columbia blood agar, Schaedler agar, and Chocolate agar. Concluding the procedure, handmade interim spacers were implanted. Articulating spacers were used for hips, whereas knees underwent implantation of static spacers, both of which were made from polymethylmethacrylate (PMMA) bone cement (Copal G + C or Palacos G + C, Heraeus medical, Wehrheim, Germany) and had 5–10% of its weight added in antibiotics based on resistance testing (usually 2 g Vancomycin per 40 g cement, 4 g of Meropenem for gram negatives, 600 mg of Voriconazole, or 200 mg of Amphotericin for fungal organisms). In some individual cases, definitive resection arthroplasty of the hip (Girdlestone) was performed, usually if it was likely prior to first-stage surgery that the patient did not wish for any further surgeries due to limited life expectancy, cognitive disorders, or severe comorbidities.

Joint aspiration was not routinely performed before reimplantation given the expected poor sensitivity and specificity [22]. Systemic antibiotic therapy based on the infecting organism was administered for at least two weeks intravenously, followed by four weeks of oral treatment. If inflammatory serum markers (C-reactive protein and interleukin 6) had declined and soft tissues were healed, reimplantation was planned [23]. Eradication of infection was defined based on the criteria defined by Diaz-Ledezma et al. [19] citing healed wounds, absence of PJI-related mortality, and no further revision surgery for infection. For early (within 4 weeks after the last surgery) or acute infections, a DAIR procedure was performed, which includes irrigation, debridement, antibiotic therapy, and the retention of the implant with component exchange, while for later, chronic infections or after a failed debridement procedure, removal of the prosthesis and a repeat staged revision was recommended.

The implants for reimplantation were chosen in consideration of individual factors, such as age, defect size, and bone quality. In three cases, an arthrodesis implant was selected for the second-stage reimplantation. During the reimplantation surgery, deep tissue samples were obtained and sent for microbiological analysis. After reimplantation was completed, antibiotics were administered for 2 weeks in case of negative intraoperative culture results, and for six weeks if positive cultures at the time of reimplantation were obtained.

### Statistical analysis

We pseudonymized all patient data before conducting any statistical analysis.

Descriptive statistics were investigated for data distribution and categorical variables. Means and ranges are used to report parametric data, whereas non-parametric data are displayed using medians and interquartile ranges (IQR). Differences in groups of binary variables were compared using Fisher’s exact test, whereas metric variables were compared using the Mann–Whitney *U* test for non-parametric distributions or student’s *t* test for parametric distributions of data. Re-reinfection, i.e., failure of the repeat two-stage exchange arthroplasty defined by the Delphi-based criteria [19], was set as the primary outcome measure. Secondary outcome measures were as follows: completion of the repeat two-stage exchange arthroplasty with reimplantation, and amputation.

Implant survival was assessed using the Kaplan–Meier survival analysis with 95% confidence intervals (CI) presented [24]. Differences in survival were compared using the log-rank test [25]. Again, primary endpoint was the diagnosis of re-reinfection in line with the Delphi consensus criteria [19].

All tests were two tailed with an alpha level of 5% considered significant.

## Results

### Reimplantation rates

Among the 55 patients who underwent the first stage of a planned repeat two-stage exchange, five knee patients (16%, 5/32) and seven hip patients (30%, 7/23) did not complete the two-stage procedure with reimplantation of the prosthesis, making a total of twelve patients (22%, 12/55) who did not undergo reimplantation. Among these, six are considered reinfected or persistently infected, while in the other six patients the infection is considered eradicated.

Of the six patients who remained infection-free after the explantation stage, two knee patients have died of unrelated cause before the planned reimplantation surgery and four hip patients were successfully treated with a Girdlestone resection arthroplasty due to low functional demand and high surgical risk and remained infection-free until the last follow-up.

Among the patients with persisting infection or reinfection, three patients (two knees, one hip) underwent amputation, one hip patient died from sepsis that was most likely PJI related, one knee patient who was treated with Girdlestone resection arthroplasty underwent a subsequent debridement procedure due to deep tissue reinfection, and one patient with a retained hip spacer had recurrent wound infection.

Patients who did not undergo second-stage reimplantation had a higher median age-adjusted CCI (5 vs. 3 (*p* = 0.034)). Furthermore, patients with chronic kidney disease (CKD) (*p* = 0.068) and patients with a shorter time between the first two-stage and reinfection (*p* = 0.061) appeared to be at increased risk to not undergo reimplantation, although no statistical significance could be ascertained with the numbers available.

### Additional surgical performances

 25% (14/55) of patients (8 knees, 6 hips) underwent debridement, irrigation and exchange of the mobile and modular implant components prior to an eventual repeat implant removal. These patients, however, did not have a different outcome than the patients who had not undergone an additional DAIR procedure regarding the rate of re-reinfection after repeat two-stage exchange (*p* = 0.547) or the length of infection-free implant survival (*p* = 0.750).

During the prosthesis-free interval of the repeat two-stage procedure, ten patients (4 knees, 6 hips) underwent a singular spacer exchange procedure and one hip patient underwent three consecutive spacer exchange procedures, resulting in a total of 20% of patients (11/55) having undergone at least one spacer exchange. The reason for a spacer exchange was spacer dislocation in four cases and clinical signs of persisting infection in nine cases. In our hospital, a spacer exchange due to mechanical reasons, like dislocation, was indicated if the spacer caused pain or disability, threatened neurovascular structures, or could potentially cause skin necrosis, analog to the criteria postulated at the International Consensus Meeting in 2018 [26, 27]. Spacer exchange due to persisting infection was indicated following the Delphi-based consensus criteria [19]. While the cultures taken during spacer exchange were culture negative in 45% (5/11) of spacer exchanges, a different, new organism was cultured during 55% (6/11) procedures compared to the first-stage surgery.

### Re-reinfection rates and infection-free implant survival after reimplantation

Ultimately, 78% (43/55) of patients underwent reimplantation after a median time of 89 days (IQR 71–143). However, further revision for re-reinfection was performed in 51% (22/43) of cases (14 knees, 8 hips) after reimplantation. To treat re-reinfection, a total of 15 DAIR procedures, twelve repeat two-stage exchange procedures, and nine amputation surgeries were performed in this subgroup. One patient refused further surgical treatment of his reinfected prosthesis and thus remained with a fistula until last follow-up.

**Fig. 2:**
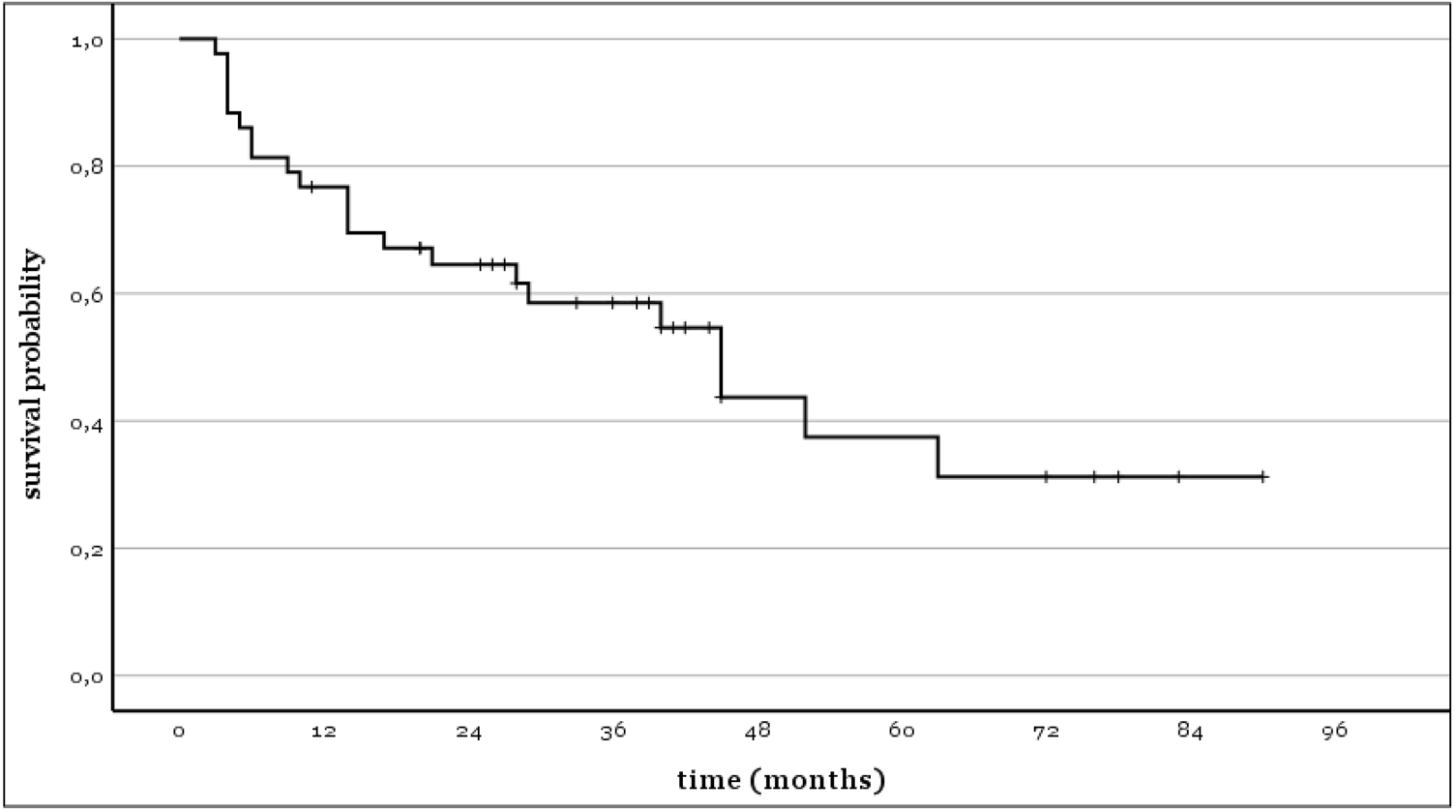
Kaplan–Meier survival curve for reinfection-free survivorship after completed repeat two-stage revision

For the 43 cases in which the repeat two-stage procedure was completed, a Kaplan–Meier analysis of the infection-free implant survival was conducted (Fig. 2). After 1 year, the cumulative infection-free survival probability was 77% (95% CI 64–89%), 65% after 2 years (95% CI 50–79%), and 38% after 5 years (95% CI 18–57%).

Patients suffering from diabetes mellitus had a significantly lower infection-free survival rate than patients without diabetes mellitus (33% (95% CI 9–57%) vs 82% (95% CI 67–96%) after 2 years, *p* < 0.001).

The infection-free survival was not different between TKA and THA (70% (95% CI 53–88%) vs 56% (95% CI 31–80%) after 2 years, *p* = 0.483).

### Overall re-reinfection rates, amputation rates, and mortality

Among all 55 patients who underwent first-stage resection arthroplasty, 51% (28/55) ultimately suffered from re-reinfection. This includes seven amputation surgeries in the subgroup of knee PJI (22%, 7/32) and two amputations that were performed on hip patients (9%, 2/23), resulting in a total amputation rate of 16% (9/55) in our cohort. The overall rate of re-reinfection was not different between hip (48%, 11/23) and knee (53%, 17/32) arthroplasties (*p* = 0.787). However, the rate of re-reinfection was significantly higher in obese patients (body mass index ≥ 30 kg/m^2^) (*p* = 0.026).

Overall, 29% (16/55) of patients have died during the follow-up period after a mean time of 32 months (range 1–92). One-year mortality was 11% (6/55) and 30-day mortality was 4% (2/55).

### Aseptic complications

Aseptic complications after the repeat two-stage exchange arthroplasty occurred in seven cases: two patients underwent revision for aseptic TKA loosening, one femoral and tibial each (8 and 10 months postoperatively), and five patients suffered hip dislocation and underwent closed reduction in all cases at a median follow-up of 3 months.

### Microbiological findings

The microbiological findings at the explantation stage of the first and the repeat two-stage exchange surgeries and at re-reinfection are presented in Table 3.

**Table 3:** Causative organisms at each infection

Organism	First two-stage exchange	Second two-stage exchange	Re-reinfection
Coagulase-negative s*taphylococci*	17 (31%)	14 (25%)	5 (18%)
*Staphylococcus aureus*	10 (18%)	4 (7%)	4 (14%)
*Streptococci*	4 (7%)	4 (7%)	1 (4%)
Gram-negative bacteria	3 (5%)	10 (18%)	5 (18%)
*Candida spp.*	0 (0%)	1 (2%)	0 (0%)
*Enterococci*	0 (0%)	1 (2%)	1 (4%)
Anaerobic	3 (5%)	0 (0%)	1 (4%)
Culture-negative	6 (11%)	6 (11%)	1 (4%)
Polymicrobial	10 (18%)	15 (27%)	9 (32%)
Missing	2 (4%)	0 (0%)	1 (4%)
Total	55 (100%)	55 (100%)	28 (100%)

While coagulase-negative staphylococci, mostly *Staphylococcus epidermidis*, were the most common finding during surgery for the first episode of PJI (31%, 17/55), polymicrobial findings increased with the second and third PJI, being the most reported microbiological finding at reinfection (27%, 15/55) and re-reinfection (32%, 9/28). However, with the numbers available, there was no difference in polymicrobial infections between the first, second, and third PJI (*p* = 0.320).

In 9% (5/55) of all cases (two knees, three hips), the microorganism isolated at the explantation stage of the first two-stage exchange was persistent at the explantation stage of the second two-stage exchange. 67% (37/55) of reinfections were caused by a new pathogen that had not been isolated at the explantation stage of the first two-stage exchange. 24% (13/55) of the patients in this study cohort were culture negative at either one or both two-stage exchanges or, in two cases, had missing information about the microbiological findings at the first two-stage exchange. The persistence rates among knee patients (6%, 2/32) and hip patients (13%, 3/23) do not statistically differ (*p* = 0.639).

Of all 28 patients who are considered re-reinfected after the repeat two-stage exchange arthroplasty, 68% (19/28) of re-reinfections are considered new infections. A persistent pathogen was observed in 21% (6/28) of re-reinfected patients. Two patients (7%, 2/28) were culture negative at the second two-stage exchange and one patient’s microbiological findings at re-reinfection are missing, as the diagnosis has been made at an outside hospital. Knee patients had a higher persistence rate of 24% (4/17) than hip patients with a rate of 18% (2/11), however, without a significant statistical difference between the two groups (*p* = 1.000).

28% (13/55) of patients had positive cultures during reimplantation surgery at the first episode of PJI. During the second two-stage procedure, 37% (16/43) of patients had positive cultures during reimplantation. However, this difference was not significant between the two episodes (*p* = 0.182).

With the numbers available, no microbiological factors (culture-negative infection, positive culture at reimplantation, or polymicrobial infection) were associated with the risk of re-reinfection or reduced implant survivorship.

## Discussion

Two-stage exchange arthroplasty remains the gold standard for the treatment of chronic periprosthetic joint infection. Still, infection recurrence is observed in 6% to 33% of the cases [4–10] and patients suffering from reinfection often face a long course of multiple additional surgeries with a potentially poor outcome [28]. However, given the relatively low number of repeat two-stage exchange arthroplasty procedures reported, data regarding the outcome of patients undergoing this procedure are scarce. This study provides additional data about the success rate of repeat two-stage exchange procedures in a single tertiary revision arthroplasty center and identifies possible risk factors associated with further revision surgery as well as microbiological insights with a high percentage of polymicrobial infections in repeat two-stage procedures and frequent positive cultures during second-stage reimplantation. In our cohort, the probability to remain infection-free amounted to only around 65% after 2 years and more than half the patients in this cohort ultimately suffered from re-reinfection following this procedure. We found that obese patients and diabetics were at increased risk for further infection and reduced infection-free implant survival, respectively, and patients with a higher comorbidity score were more likely not to undergo second-stage reimplantation.

Prior studies have reported re-reinfection rates after repeat two-stage exchange arthroplasty ranging from 22.2 to 49% [11–15]. While the comparability to previous studies is somewhat limited by different demographic and surgical details of the patients included, the failure rate presented in our study is relatively high. It should be noted that surgeons must consider various factors in the success of two-stage procedures that are difficult to account for in the retrospective study designs of our and related studies.

Kheir et al. investigated 60 patients who underwent further surgical intervention after a failed two-stage exchange of infected THA or TKA, including 26 patients, with a repeat two-stage procedure that was successful in 62% of patients [11]. The authors note a higher risk for reinfection after a debridement procedure with retention of the prosthesis compared to a repeat two-stage procedure and propose a more aggressive approach with repeat staged revision. While we also noted that 25% of patients who ultimately underwent a repeat two-stage procedure had a failed attempt to retain the implant, they did not have a worse outcome after the two-stage procedure. Nonetheless, considering that a DAIR procedure is far less invasive than a complete implant removal, we suggest that if early (within 4 weeks) or acute reinfection occurs, patients should be counseled regarding the high risk of failure after a DAIR procedure, but if soft tissue conditions and the patients general state allows for it, it can be an option to avoid further staged revision.

Khan et al. investigated repeat two-stage procedures of infected THA and reported a success rate of 57% at two-year follow-up in 42 patients, which is comparable with our study results [12]. With 33% of patients deceased before the two-year follow-up, the mortality presented in their cohort also corresponds with our results. Khan et al. concluded that an underlying host problem might be the cause for poor outcome regardless of treatment modality and recommended further research on host optimization and other treatment methods for repeat PJI. Considering the equally poor results of repeat two-stage exchanges in our cohort, we agree that patients should be informed regarding this dismal perspective and a mutual decision should be made if further staged revision is planned.

Considering the poor outcome and high risk for re-revision in repeat two-stage procedures in our and the aforementioned studies, there is an urgent need for further investigation on alternative treatment options for repeat PJI.

The retention of a spacer can be a good option in selected patients [29, 30]. Nevertheless, a risk for recurrent infection should be taken into account, particularly if one considers that in the present study, even if no reimplantation is performed, there is a high risk of further revision surgeries for infection.

Amputation is considered a last resort treatment that is usually chosen if the infection cannot be controlled with any other surgical or medical treatment option. The possibility of curing the infection comes at the cost of low functional status and high mortality [31–33]. Nevertheless, amputation can still yield good patient satisfaction [33].

With Girdlestone resection arthroplasty, partial limb function can be preserved, while the infected prosthesis or spacer is removed. However, the rates of complication, mortality, and reoperation are reported to be high [34]. On the other hand, a conversion back to a THA can achieve satisfactory results, if the patient is deemed suitable [35]. This also applies to arthrodesis of the knee [35]. Wu et al. recommended performing arthrodesis as the treatment of choice after a failed two-stage exchange of TKA [36]. In our cohort, only three patients were treated with an arthrodesis instead of a second stage reimplantation and all three subsequently suffered from re-reinfection. Because of the low occurrence in our study, however, we cannot make any general statement concerning the outcome after knee arthrodesis.

Chronic antibiotic suppression can be an option for high surgical risk patients or in cases with large bone defects in which reconstruction would be likely to fail [35]. However, surgeons must consider long-term side effects of suppression therapy and the risk of uncontrolled infection. Furthermore, Leitner et al. investigated the mortality rate of patients with these salvage procedures and found a long-term mortality of 44% at a median follow-up of 8 years. They recommended a chronic fistula instead of further resection arthroplasty [37]. Regarding repeated revisions in TKA, they recommended considering amputation early after multiple failed revision arthroplasties [37]. Surgeons must always consider that these patients can have a very limited life expectancy and high mortality and further surgical intervention must be weighed regarding the high risk of failure. Nevertheless, if a repeat two-stage exchange for PJI is planned, these patients should also be counseled regarding alternative approaches, but further research is much needed to determine the value of different treatment modalities with regard to reinfection rates and quality of life.

In order to potentially optimize host factors and assess individual risk, patient- and procedure-related predisposing factors are relevant. However, to our knowledge there are no studies that specifically investigate risk factors in the setting of a repeat two-stage exchange. In the present study, diabetics had a lower implant survival probability. Although this factor has not been described for repeat two-stage revisions, diabetes mellitus is a known risk factor for infection of total hip or knee arthroplasty [38]. Additionally, it has been shown that poorly controlled diabetes appears to be linked to even worse outcomes [39] and in many cases diabetes and obesity are connected [40]. Considering that Fehring et al. reported an increase of obese patients from 30.4% in 1990 to 52.1% in 2005 [41], not only the risk for infection after primary arthroplasty but also the risk for further infections must be expected to increase if re-revision is performed. It appears warranted to emphasize diabetes control and to address related complications, such as foot ulcers early on as they might pose as a source of PJI [42]. Future studies should investigate the effect of potential host optimization on the outcome of two-stage procedures and repeat revision.

Polymicrobial infections were most common in patients with repeat two-stage revision but were not associated with a worse outcome. However, the high prevalence of polymicrobial infections must be taken into account to plan the systemic treatment. Contrary to this, Tan et al. found that polymicrobial PJI has a worse outcome and higher PJI-related mortality than PJI caused by one organism or culture-negative PJI, although this was done in patients with first PJI [43]. In our study cohort, most of the patients suffering re-reinfection after the repeat two-stage exchange arthroplasty presented with a new pathogen. Zmistowski et al. suggested that especially patients with poor health status and a high number of comorbidities are at risk for reinfection with new organisms because of their higher disposition to infections rather than due to a failed treatment [21]. However, we found that in this study the percentage of persisting infections increased from the second PJI to re-reinfection. Zmistowski et al. found that *Staphylococcal* organisms, especially *methicillin-resistant Staphylococcus aureus*, have a high risk to be persistent in recurrent PJI. Further research on pathogen persistence rates, microbiological resistance patterns, and antimicrobial treatment could provide interesting additional insight.

This study’s findings must be interpreted considering several limitations: Our study data were collected at a single institution and therefore might include bias that comes from local preferences or routines. There may have also been a selection bias since our study cohort only consists of patients who were deemed fit enough for a repeat two-stage exchange arthroplasty; however, in our practice generally further surgery was recommended if reinfection was present. Nonetheless, it is possible that patients had undergone surgery elsewhere or that suppression treatment has been initiated. Furthermore, for some statistical analysis there might be sparse data bias as the number of risk factors and number of events are limited and a multivariate analysis is not helpful in this situation. Nevertheless, we think that our findings are a valuable addition to the existing research on this rare issue.

## Conclusion

Repeat two-stage revision for recalcitrant infection after a two-stage procedure for PJI leads to further infection in around 50% of patients at mid-term follow-up. Particularly, obese patients and diabetics are at high risk of reinfection. Surgeons should be aware of the high percentage of polymicrobial infections encountered at a repeat two-stage procedure and plan antibiotic treatment accordingly. Furthermore, particularly in comorbid patients an additional share of patients did not reach second-stage reimplantation; therefore, alternative strategies should be discussed in order to avoid re-revisions in this challenging group of patients.

## Data Availability

The datasets used and/or analyzed during the current study are available from the corresponding author upon reasonable request.
